# Identification and Functional Characterization of the Gene Cluster Responsible for Fusaproliferin Biosynthesis in *Fusarium proliferatum*

**DOI:** 10.3390/toxins13070468

**Published:** 2021-07-06

**Authors:** Asja Ćeranić, Thomas Svoboda, Franz Berthiller, Michael Sulyok, Jonathan Matthew Samson, Ulrich Güldener, Rainer Schuhmacher, Gerhard Adam

**Affiliations:** 1 Institute of Bioanalytics and Agro-Metabolomics, Department of Agrobiotechnology (IFA-Tulln), University of Natural Resources and Life Sciences, Vienna (BOKU), Konrad-Lorenz-Str. 20, 3430 Tulln, Austria; asja.ceranic@boku.ac.at (A.Ć.); franz.berthiller@boku.ac.at (F.B.); michael.sulyok@boku.ac.at (M.S.); jonathan.samson@boku.ac.at (J.M.S.); rainer.schuhmacher@boku.ac.at (R.S.); 2 Institute of Microbial Genetics, Department of Applied Genetics and Cell Biology, University of Natural Resources and Life Sciences, Vienna (BOKU), Konrad-Lorenz-Str. 24, 3430 Tulln, Austria; thomas.svoboda@students.boku.ac.at; 3 School of Life Sciences, Technische Universität München, Maximus-von-Imhof-Forum 3, Weihenstephan, D-85354 Freising, Germany; u.gueldener@tum.de

**Keywords:** fusaproliferin, terpestacin, preterpestacin, emerging mycotoxins, knock-out, terpenoid synthase, cytochrome P450 oxidoreductase, FAD-oxidase, acetyltransferase

## Abstract

The emerging mycotoxin fusaproliferin is produced by *Fusarium proliferatum* and other related *Fusarium* species. Several fungi from other taxonomic groups were also reported to produce fusaproliferin or the deacetylated derivative, known as siccanol or terpestacin. Here, we describe the identification and functional characterization of the *Fusarium proliferatum* genes encoding the fusaproliferin biosynthetic enzymes: a terpenoid synthase, two cytochrome P450s, a FAD-oxidase and an acetyltransferase. With the exception of one gene encoding a CYP450 (*FUP2*, FPRN_05484), knock-out mutants of the candidate genes could be generated, and the production of fusaproliferin and intermediates was tested by LC-MS/MS. Inactivation of the *FUP1* (FPRN_05485) terpenoid synthase gene led to complete loss of fusaproliferin production. Disruption of a putative FAD-oxidase (*FUP4*, FPRN_05486) did not only affect oxidation of preterpestacin III to terpestacin, but also of new side products (11-oxo-preterpstacin and terpestacin aldehyde). In the knock-out strains lacking the predicted acetyltransferase (*FUP5*, FPRN_05487) fusaproliferin was no longer formed, but terpestacin was found at elevated levels. A model for the biosynthesis of fusaproliferin and of novel derivatives found in mutants is presented.

## 1. Introduction

*Fusarium* mycotoxins are a major issue for food and feed safety. Due to climate change, the pathogen spectrum is expected to change, potentially leading to an increased abundance of “emerging mycotoxins” in unusual places [1]. For example, besides well-known Fusarium mycotoxins, such as trichothecenes, zearalenone and fumonisins, for which legal limits have been enacted, additional metabolites are produced by various *Fusarium* species, including the emerging mycotoxin fusaproliferin (FUP). Due to the lack of commercially available standards, the data on the occurrence of FUP and its deacetylated derivative terpestacin in food and feed products are still very limited. FUP concentrations as high as 19.6 mg/kg have been reported for rice samples from Morocco (for review see [2]). Preharvest maize ears from Italy collected in 1994 contained up to 500 mg/kg of this mycotoxin [3]. *F. proliferatum* or *F. subglutinans* isolates from maize kernels from Iowa [4] produced up to 1 g/kg FUP in vitro. Extremely high FUP production of up to 50 g/kg on irradiated maize was reported for a *F. temperatum* strain from Argentina under optimal conditions [5].

FUP belongs to the class of bicyclic sesterterpenoids and was purified and characterized for the first time from maize kernels inoculated with *Fusarium proliferatum* [6]. The deacetylated derivative has a complex history due to a confusion in the older literature regarding its stereochemistry. Terpestacin was isolated for the first time in the early 1990´s from *Arthrinium* strain FA1744 (*Sordariomycetes*, *Xylariomycetidae*) as an inhibitor of syncytium formation induced by the human immunodeficiency virus (HIV) in infected human cells [7]. In 1998, the total enantiospecific synthesis of (+)-terpestacin was reported [8]. However, in 2001 Gräfe and co-workers isolated (−)-terpestacin from the fungus *Ulocladium* sp. HKI 0226 (*Pezizomycotina*, *Dothideomycetes*) [9]. *Bipolaris sorokiniana* (*Pezizomycotina*, *Dothideomycetes*) was reported to synthesize siccanol, which was initially designated to be “11-epi”-terpestacin [10]. However, as summarized by [11], siccanol is the same compound as (−)-terpestacin and is just deacetylated FUP [12].

Data concerning toxicity and mode of action of FUP are still limited. FUP has been isolated based on its toxicity in brine shrimp larvae with an LD_50_ of 53.4 µM [13]. Variable cytotoxicity in the low µM range was observed with different mammalian cell lines [13,14,15]. Severe teratogenic effects were reported in chicken embryos, e.g., cephalic dichotomy, macrocephaly, and limb asymmetry in 20% of the embryos exposed to an extremely high level (5 mM) of FUP [3]. This might be due to the non-covalent interaction of FUP with DNA [16]. Jung, et al. [17] have isolated terpestacin from *Embellisia chlamydospora* (*Pezizomycotina*, *Dothideomycetes*) based on its ability to inhibit angiogenesis in bovine aortic endothelial cells at doses that are not cytotoxic. In a phage display approach, biotinylated terpestacin was specifically bound to an 81-amino-acid fragment of the human ubiquinol-cytochrome c reductase binding protein (UQCRB) in mitochondria, which is, therefore, the proposed target of terpestacin (and presumably FUP) in mammalian cells [18].

FUP and terpestacin are also clearly phytotoxic. The root growth of Italian ryegrass seedlings was strongly inhibited by 100 mg/L (~250 µM) terpestacin [10]. FUP (at 35 mg/L) has been described to cause structural changes in chloroplasts of maize plants, including thylakoid disorganization and severe damage to the outer chloroplast membrane [19]. More recently, due to its phytotoxicity towards the weedy *Bromus tectorum* (cheatgrass), it was suggested that terpestacin might be involved in the pathogenicity of the fungus *Rutstroemia capillus-albis* (*Sordariomyceta*) [20]. Terpestacin was recently also found as a phytotoxic compound of *Eufusicoccum batangarum*, causing scabby canker of *Opuntia* [21]. 

Yet, with respect to food and feed safety, the most relevant producer of FUP are *Fusarium* species, such as *F. temperatum* and *F. subglutinans,* and particularly *F. proliferatum* on maize. Genome sequences have been reported [22] for two *F. proliferatum* strains, NRRL 62905 (isolated from maize with ear rot in Iowa, [4]) and ET1 (an auxin-producing strain isolated from an orchid [23]). In a previous study on secondary metabolism genes in the *Fusarium fujikuroi* species complex, no candidate genes for fusaproliferin biosynthesis were reported (see supplementary Table S1 in reference [22]). FUP and ophiobolins both belong to the class of sesterterpenoids. In *Aspergillus clavatus* [24], several ophiobolin biosynthesis genes have already been described, including the ophiobolin synthase for the formation of the core ring structure of ophiobolins. Although it has been proposed that ophiobolins and fusaproliferin cyclases belong to different groups of sesterterpene cyclases [25], we expected that high sequence similarity might nevertheless exist. The aim of our study was to identify the genes necessary for FUP production in *F. proliferatum* and to get deeper insights into the biochemical mechanisms of its formation by analyzing gene disruption mutants using LC-MS/MS.

## 2. Results

### 2.1. Bioinformatic Identification of the Terpenoid Synthase Candidate Gene 

A BlastP search was performed using the ophiobolin F synthase *oblA* (UniProtKB-A1C8C3) from *Aspergillus clavatus* as the query sequence. This protein consists of an N-terminal part belonging to the terpene synthase family and a C-terminal part related to the FPP/GGPP synthase family. The BlastP search against the genome of *F. proliferatum* NRRL 62905 revealed only one good candidate, the product of the gene (FPRN_05485), which contains both domains and shows reasonably high similarity (only 23.1% identity in the N-terminal 320 amino acids of the terpenoid synthase domain, and 37.1% identical amino acids in the predicted C-terminal geranyl/farnesyl diphosphate synthase). Since *F. proliferatum* is not known to produce ophiobolins, we selected this gene as a candidate for further experimental analysis.

### 2.2. Functional Testing of FPRN_05485

**Figure 1:**
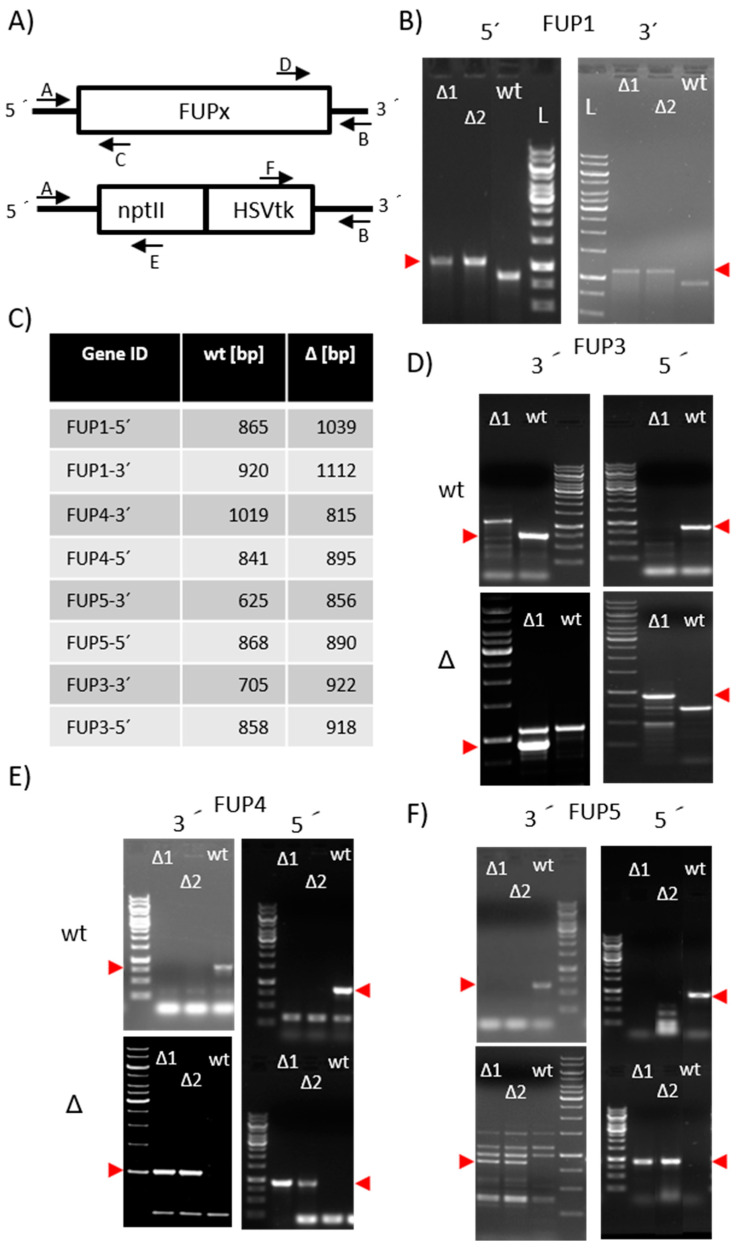
PCR confirmation of the knock-out of *FUP1* and *FUP3* to *FUP5*. (**A**) screening scheme with primer locations indicated; (**B**) screening of *FUP1* knock out using multiplex PCR (Primer A + C + E or B + D + F). The red arrows indicate the expected length of the knock-out band; (**C**) length of the expected fragments is shown in Table 2; (**D**–**F**) screening with two primers. For the samples on the top panels the wild-type primer pair (A + C or B + D) was used, on the bottom the pair specific for the knock-out (A + E or B + F) was used; (**D**) screening for *FUP3* knock-out (**E**) screening for *FUP4* knock out; (**F**) screening for *FUP5* knock out; (**D**–**F**) the red arrows beside the respective gel indicate the expected lengths. Size standard: 1 kb ladder (Thermo Fisher Scientific, Vienna, Austria).

To determine whether this candidate gene is involved in FUP synthesis or not, it was inactivated by homologous recombination in strain NRRL 62905, for which FUP production in vitro has been previously described [4]. Upstream and downstream regions were cloned into the vector pKT245 [26], flanking the G418 resistance cassette. For the disruption, the 5´ and the 3´ flanking regions were amplified together with overlapping parts of the neomycin phosphotransferase (*nptII*) gene. Transformants, where the split selection marker was restored by homologous recombination, were selected on 30 mg/L G418 and screened using multiplex PCR with three primers, one located outside of the flanking region, one in the resistance cassette and one in the target gene (see Figure 1A). Fragment lengths allowed to determine whether the resistance cassette was integrated at the desired locus or ectopically. Only two out of 23 transformants (8.7%) produced the fragment lengths indicating that the gene was replaced successfully (Figure 1B,C). The single conidia purified knock-out strains, designated FUP1Δ1 (strain #2272) and FUP1Δ2 (#2273), were used for testing FUP production on autoclaved rice. While extracts of two wild-type strains (inoculated from different plates of NRRL 62905, each in three repeats) contained 39 ± 13 mg/L and 49 ± 8 mg/L FUP, no FUP was detected in the two knock-out mutants, which is in agreement with our hypothesis that FPRN_05485 encodes the terpenoid synthase for the first step of fusaproliferin biosynthesis. This gene was, therefore, designated as *FUP1*.

### 2.3. Functional Analysis of the FUP Gene Cluster

Based on the finding that FPRN_05485 is responsible for the formation of the basic backbone structure of FUP, we took a closer look at neighboring genes and deactivated other putative members of the FUP gene cluster. Both sequenced *F. proliferatum* strains contain a highly collinear cluster with very similar predicted proteins, and the putative FUP genes were designated as listed in Table 1. The two putative transcription factors (*FUP6* and *FUP7* in parentheses) are the subject of a separate study and still speculative. The putative secondary metabolite cluster is flanked by housekeeping genes, a predicted prolyl-tRNA synthetase and lactate/malate dehydrogenase, respectively. For the putative prolyl-tRNA synthetase, different N-termini are predicted in the two strains, leading to the low sequence similarity (Table 1).

While the work presented here was well advanced, the gene cluster for terpestacin production in *Bipolaris maydis*/*Cochliobolus heterostrophus* was reported and the pathway was reconstituted in *Aspergillus oryzae* [27]. The cluster described is fully conserved in *F. proliferatum* (Table 1).

Upstream of the terpenoid synthase (TPS) is a gene with high similarity to cytochrome P450 oxidoreductases (CYP-1), which we designated *FUP2*. A second CYP450 gene (CYP-2), encoded by FPRN_05488, was designated *FUP3*. The two encoded proteins are expected to introduce the hydroxy groups at C_16,_ C_17_, and C_24_, which can presumably be further metabolized. *FUP4* might be an FAD-dependent oxidoreductase (prediction: FAD-binding protein) expected to catalyse the formation of the C_16_ keto-group. The addition of the acetyl group to the C_24_-OH is supposed to be catalyzed by *FUP5* (FPRN_05487), which shows 27% identical amino acids and 45% similarity (E-value 1E^−12^) to an acetyltransferase of *Aspergillus nidulans* (Sequence ID: A0A1U8QYW8.1).

#### 2.3.1. Preparation of Knock-Out Strains

To test the proposed role in FUP biosynthesis we started to generate gene disruption mutants (see Figure 1). Plasmids for transformation were constructed as described in Material and Methods. Primers used for the amplification of the flanking regions of the candidate genes that were cloned into the disruption vector are listed in Table 2. 

Primer pairs used for screening of transformants (as indicated in Figure 1) are listed in Table 3. The common primers located in the resistance cassettes are given in Material and Methods.

The screening was performed by PCR according to the scheme shown in Figure 1A One outer primer (A or B) were used in the PCR mixes together with each a primer located inside the gene to be disrupted (C or D) and one located in the resistance cassette (E or F). The diagnostic gels show the confirmation of the respective disruption (Figure 1B–F). 

The disruption of *FUP2*, one of the cytochrome P450 genes, was attempted in four independent transformation experiments. Although a total of 178 G418-resistant transformants were obtained and analyzed, none of them contained the desired gene disruption. In comparison, during the disruption of *FUP4* (putative FAD-oxidoreductase), we obtained two transformants out of 62 (3.2%) exhibiting PCR fragments of the right length at the 5´- and the 3´-flanking region. We also obtained two transformants (out of 63 = 3.1%) where the putative acetyltransferase *FUP5* was replaced by the resistance cassette. The disruption of the second CYP450 gene, *FUP3*, was successful as well, however, only one correct transformant (out of 55 = 3.6%) was obtained. Single-conidia purified “second generation” isolates of transformants were used for toxin production tests on autoclaved rice. After two weeks of incubation at 20 °C in the dark, the samples were extracted and analyzed by semi-targeted LC-MS/MS. 

#### 2.3.2. Search for Predicted FUP Intermediates and Structurally Related Compounds

The extracts of different knock-out strains were analyzed by LC-HRMS with the aim to search for the presence of biosynthetic intermediates of the FUP biosynthetic pathway. A list of putative intermediates, to be confirmed in the following evaluation steps, was generated, including six of those proposed by Narita et al. [27]. Amongst them, terpestacin and FUP were confirmed with purified standards, while unique retention time values were assigned to other putative intermediates when more than two ion species (calculated from the sum formula of the intermediate) were found at a single retention time. Consequently, those candidates that fit to sum formulae of preterpestacin I, II (a, c) and III were included in the target list. The list was extended by including expected structurally related compounds (i.e., derived by oxidation and acetylation). While acetyl-derivatives were not found (with the exception of FUP, the acetyl derivative of terpestacin), we were able to extend the list with several putative oxidation products (i.e., preterpestacin II b, terpestacin, terpestacin aldehyde and oxo-preterpestacin I). In total, eleven compounds were considered (of which two were confirmed by standards plus nine further suspects were assigned based on observed masses) (Table 4).

**Figure 2:**
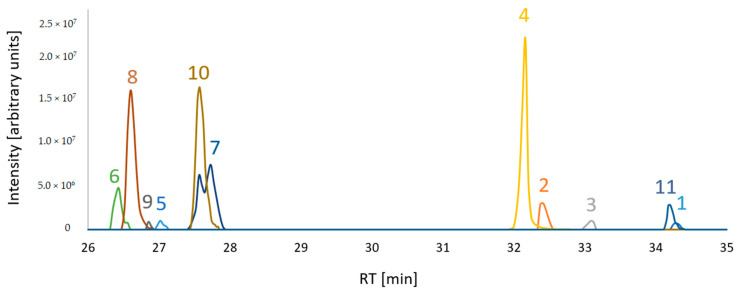
Extracted ion chromatograms of putative FUP biosynthetic pathway compounds from a sample mixture which consisted of equal volume aliquots of each sample type. [M+H]+ adducts were considered for compounds 1, 2, 3, 8, 9 and [M-H_2_O+H]+ for 4, 5, 6, 7, 10, 11. For visualization of all compounds in a single overlaid chromatogram, the peak abundances of 8, 4 and 11 are divided by 10.

A mixture of samples from wild-type and the knock-out mutants was prepared to test whether evidence for the occurrence can be obtained. The result is shown in Figure 2.

#### 2.3.3. Abundance of FUP and Pathway Precursors in Knock-Out Strains

**Figure 3:**
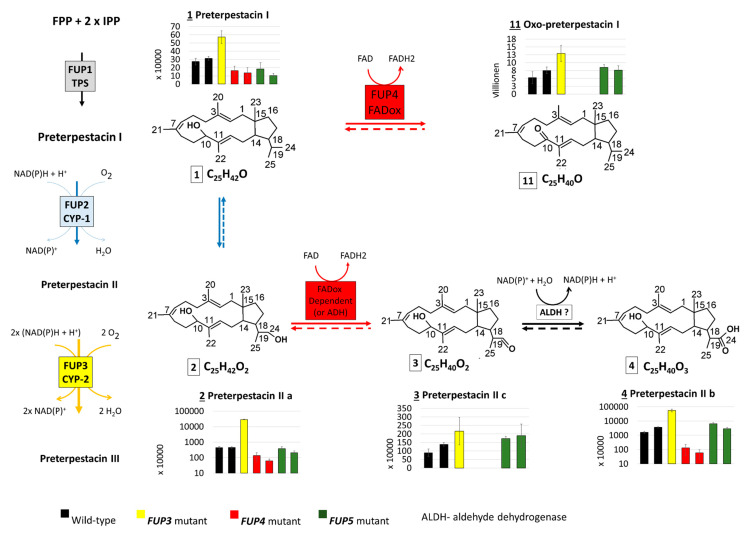

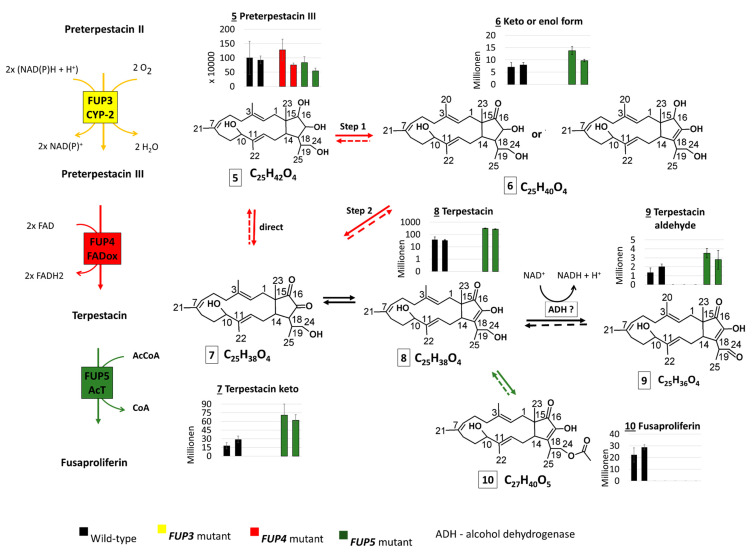
Scheme which shows the intermediates of the proposed FUP biosynthetic pathway with side reactions of pathway intermediates, as well as their abundance distribution in samples of *Fusarium proliferatum* wild-type (WT) and knock-out strains. Thick arrows correspond to reactions of the proposed pathway, thin arrows to side reactions of the pathway intermediates. Dashed arrows indicate reactions that may take place but were not proved with the present experiment.

The abundances of the confirmed and suspect compounds (from Table 4) were examined in the extracts of rice cultures of *F. proliferatum* wild-type and knock-out strains lacking enzymes from the proposed FUP biosynthetic pathway. The abundance distribution of each compound in the analyzed samples is presented with bar charts together with the proposed genes encoding the respective enzymes (Figure 3).

The end product, FUP (compound 10), could not be detected in any of the mutants with disrupted *FUP3*, *FUP4*, or *FUP5*, but was present in cultures of both wild-type strains in high amounts. These results show that all three enzymes are needed for FUP biosynthesis (Figure 3).

Terpestacin (compound 8) was found at significantly higher levels (*p* < 0.01) in both mutants with the disrupted acetyltransferase gene *FUP5*, containing about 8-fold higher abundances than the wild-type. This is in agreement with the hypothesis that *FUP5* encodes the enzyme acetylating terpestacin to fusaproliferin (Figure 3). As expected, terpestacin was lacking in mutants blocked in earlier steps of the pathway, in mutants deficient in *FUP3* and *FUP4*. Three more compounds from the suspect list (Table 4) share an abundance distribution across the samples similar to that of terpestacin. Two of them were annotated as the enol (7) and oxidized (9) forms of terpestacin, while the third compound (6) is annotated with two possible structures, one of which might be the reduced form of terpestacin or an oxidized form of preterpestacin III.

The *FUP4* gene product shows sequence similarity to FAD-oxidases and is expected to be involved in the oxidation of preterpestacin III (5) to terpestacin (8). The reaction might occur in two steps via compound 6 (one of the structure annotations) and 7 or directly over compound 7, as all compounds together with terpestacin show a similar profile across analyzed samples.

With the exception of the single available *FUP3* mutant (FPRN_05488), the annotated preterpestacin III was found in all analyzed samples at similar abundance levels (Figure 3). Both *FUP2* and *FUP3* are predicted to encode cytochrome P450s. Disruption of *FUP3* resulted in the accumulation of preterpestacin II a with a fold-change of 65 in *FUP3* disrupted mutants compared to wild-type, indicating its product catalyzes the formation of preterpestacin III from preterpestacin II a. Further, (3) and (4) were annotated as two oxidation products of preterpestacin II a, also indicated by their accumulation in *FUP3* disrupted mutants. While preterpestacin II a and (4) are found with similar abundance distribution in all analyzed samples, compound (3) was not present in the *FUP4* (FAD-oxidoreductase) mutant.

Preterpestacin I, the presumed product of the *FUP1* gene product, was detected in all analyzed samples except the *FUP1* mutant, and (11) was annotated as its derivative. Both share a similar abundance distribution across the analyzed samples (Figure 3), with two to four times higher abundance levels in mutants than in wild-type, and with the only exception that (11) is missing in the *FUP4* (FAD-oxidoreductase) mutant. Therefore, compound (11) was annotated as the oxo derivative of preterpestacin I. 

#### 2.3.4. Structure Similarity Determination of Suspect Compounds with Identified Terpestacin and FUP via LC-HRMS/MS 

The measured compounds belong to the sesterterpene family of terpenoids and consist of a 18C-bicyclic ring structure, substituted with four additional methyl groups and one to three oxygen atoms (as hydroxy or keto groups), as well as another 3 carbon atoms in form of an isopropyl group on the C18 position of the ring. The latter was found to be potentially oxidized and/or conjugated (Figure 3). Based on their putatively shared core structure, the selected compounds are expected to have similar fragmentation patterns in the obtained LC-HRMS/MS spectra, when generated under identical fragmentation conditions. Therefore, LC-HRMS/MS of all compounds from the list (Table 4) were generated using stepped collision energies at 25, 35 and 45 eV. The identity of terpestacin (TPC) and FUP was confirmed at confidence level 1 (classification system from Blazenovic, et al. [28]) for which two orthogonal values (retention time and *m*/*z*), as well as fragmentation spectra, were queried against the corresponding standards. Since no standards were available for the further suspect compounds, their fragmentation spectra were matched against those of FUP and TPC. To this end, similarity scores were calculated based on the cosine score from GNPS’s Feature Network [29]. In this respect, protonated adducts of either intact molecules or, if not present at sufficient abundance, in-source fragments after water loss were used. Both types of protonated adducts exhibited similar fragmentation patterns when compared with the very same compound which was not the case when sodium or ammonium adducts were used.

**Figure 4:**
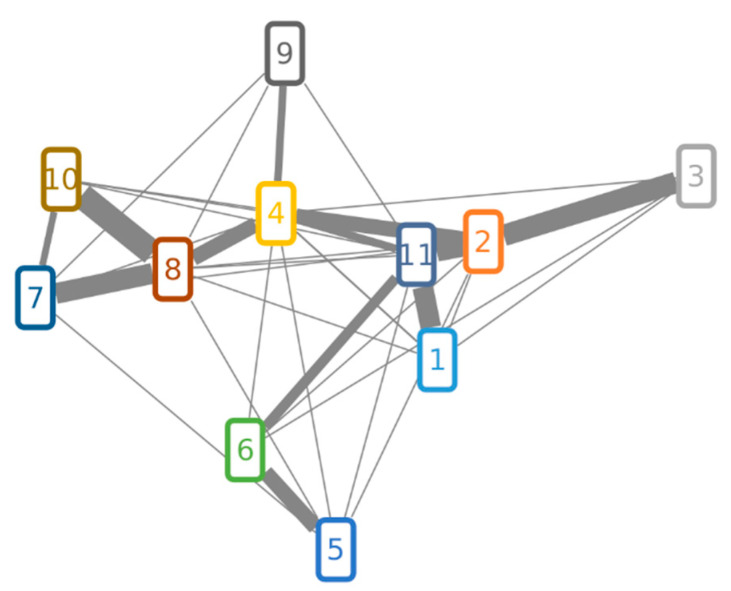
Molecular network of FUP, TPC and suspect compounds. Compound numbers in nodes correspond to those from Table 4. Two nodes are connected over the edge if their cosine score is higher than 0.5. Edges that have a cosine score higher than 0.6 have a weighted thickness that increases with the score value.

Matching of MS/MS spectra of the investigated compounds is illustrated in Figure 4. Although the cosine score shows an overall similarity between matched fragmentation spectra, some of them were more similar to each other. The highest contribution to the spectrum similarity can be attributed to fragments of the lower mass range (*m*/*z* 50 to 206), as shown in the aligned LC-MS/MS spectra (Supplementary Figures S1–S3). Highly abundant peaks of the lower mass range were also assigned to fragments of the sesterterpene core structure via MetFrag [30,31] (Supplementary Figure S4). Figure 4 shows that the MS/MS spectra of FUP (10) and the enol form of TPC (8) are more similar to each other than to other compounds, in agreement with their chemical structures only differing in an acetyl moiety. Their structural similarity is also reflected by strongly overlapping mass fragments in the higher *m*/*z* range, which is not the case with other compounds (Figures S1–S3).

## 3. Discussion

### 3.1. The Proposed Fusaproliferin Biosynthetic Pathway in F. proliferatum

While this work was in progress, the (-)-terpestacin biosynthetic pathway from *Cochliobolus heterostrophus* has been reconstituted by heterologous expression of genes in *Aspergillus oryzae* [27]. Our results are in agreement with the published model. The FUP biosynthetic pathway starts with the enzyme encoded by *FUP1* that combines a C-terminal prenyltransferase domain responsible for the synthesis of geranyl-geranyl-pyrophosphate with the N-terminal terpene cyclase domain, similarly, as described for ophiobolin synthase. The first product, preterpestacin I, is then decorated by oxygenation steps that are catalyzed by two cytochrome P450s. First, the Fup2 protein (CYP-1 in Table 1) introduces a hydroxyl group at the C24 position resulting in the formation of preterpestacin II a, which can be further oxidized. In this respect, two oxidation steps are proposed. The annotation of preterpestacin II b and II c in the measured samples suggests that the oxidation of the hydroxyl group at C24 to an aldehyde and further to a carboxylic group takes place via unspecific alcohol and aldehyde dehydrogenases, but interestingly the formation of the aldehyde is also strongly reduced in the *FUP4* mutant, lacking functional FAD-oxidoreductase. Our finding of preterpestacin II a and II c in *F. proliferatum* supports the results from Narita et al. [27].

The second P450 catalyzes the hydroxylation at C16 and C17 of preterpestacin II a, producing preterpestacin III. Subsequently, a putative FAD-dependent oxidoreductase, encoded by *FUP4*, catalyzes the oxidation of the hydroxy group at the C16 position to a keto group, leading to the formation of (-)-terpestacin, which is the immediate precursor of FUP. The identification of the oxo-derivative of preterpestacin I (compound 11) suggests that preterpestacin I might also serve as a substrate of the Fup4 FAD-oxididoreductase. Two side products of terpestacin could be further annotated. Similar to preterpestacin IIa, our results suggest that terpestacin may be oxidized to the corresponding aldehyde (compound 9) via unspecific alcohol dehydrogenases. The second presumed side product of terpestacin is compound 6 (one possible structure annotation), which might represent a reduced form of terpestacin based on its sum formula and its abundance similarity to terpestacin in the analyzed samples, and is putatively formed via the catalytic reduction by (unknown) aldo-keto reductases [32,33]. As expected, since the Fup4 protein is necessary for the formation of terpestacin, all these derivatives are absent in the *FUP4* disrupted mutant. The final step in the proposed biosynthetic pathway is the addition of an acetyl group at the C24 position of terpestacin, which is catalyzed by the acetyltransferase FUP5 (see scheme Figure 3). This enzyme seems to be quite specific, as no other acetylated precursors or derivatives were found. 

### 3.2. Possible Reasons for the Inability to Obtain a FUP2 (FPRN_05484) Mutant

In four independent attempts, the disruption of the cytochrome P450 (*FUP2*, CYP-1)) was not successful. In total, 178 transformants were analyzed; however, none of them exhibited the diagnostic fragments indicating a successful disruption. The rate of homologous recombination events for other genes in this cluster was higher than 3%, so assuming this frequency also applies to *FUP2*, it is still possible that our result is solely due to bad luck, but this is rather unlikely (chance 0.44%). In *Fusarium graminearum*, a genome-wide deletion mutant set was prepared covering 102 cytochrome P450s. Seventeen of these P450s knock-out mutants could not be generated in three attempts, which was taken as evidence that the knock out of the respective gene might be lethal [34]. While some P450s are essential since they are required for universal biochemical functions such as ergosterol biosynthesis, such a scenario seems more than unlikely here. The P450 encoded by *FUP2* should then have an essential role besides its function in the biosynthesis of the dispensable secondary metabolite FUP. A more likely scenario is that disruption of the *FUP2* might lead to the accumulation of high levels of a toxic intermediate. It is unknown whether preterpestacin I is toxic for fungi. In line with this hypothesis, in their reconstitution approach, Narita et al. [27] did not report single transformants of *A. oryzae* expressing only the terpenoid synthase gene and did not isolate preterpestacin I. Only transformants which contained both the terpenoid synthase (tpcA, the homolog of *FUP1*) and the cytochrome P450 (tpcB, corresponding to *FUP2*) or more genes of the pathway were described [27]. On the other hand, preterpestacin I was detected by LC-MS/MS in this study in the wild-type and pathway mutants other than *fup1*. A possible explanation could be that the concentrations of the biosynthetic intermediate might be below a critical threshold, while it would accumulate to toxic levels in the *FUP2* mutant, which was therefore not obtained. As shown in Figure 3, the peak areas of preterpestacin I (compound 1) are approximately two orders of magnitude below the peak area of FUP. Yet, due to different ionization properties, this cannot be directly related to concentrations. Potentially, a trivial explanation could also be that existing very slow-growing (self-poisoning) transformants were not picked and escaped further analysis.

### 3.3. Significantly Higher Terpestacin in the fup5 Mutant Strain

Peak areas for terpestacin were significantly higher in the strains lacking the acetyl transferase encoded by *FUP5* compared to the wild-type strain. FUP has been reported to be toxic for various fungi. The growth of *Alternaria brassicicola*, *Botrytis cinerea* and *Fusarium graminearum* was reduced by 38%, 19% and 30%, respectively, in the presence of FUP while, in presence of equimolar levels of terpestacin, the growth was reduced by 18%, 9% and 36%, respectively [35]. These data indicate that both toxins have a negative effect on fungal growth. Comparing the inhibiting effect between the tested fungi, *F. graminearum* shows a similar or slightly higher sensitivity towards terpestacin, while the growth of *A. brassicicola* and *B. cinerea* was stronger affected by FUP. For FUP a role of in saprophytic fungus-fungus interaction, by inhibiting other competitors, is conceivable, and also its role as a phytotoxin cannot be dismissed, as already stated in the introduction. Currently, only very limited information is available regarding the phytotoxicity of FUP and its deacetylated derivative terpestacin. Nihashi et al. [10] reported that terpestacin was more toxic than FUP for Italian ryegrass. In contrast, in a recent report it was shown that terpestacin and fusaproliferin (obtained from *Phoma exigua* causing foliar disease of oleander) inhibited germination of *Phelipanche ramosa* (*Orobanchaceae*) seeds by 25% and 40%, respectively [36].

### 3.4. Perspectives and Open Questions

The question as to whether FUP is a virulence factor of *F. proliferatum* can be tested in future work with deletion mutants. The evidence for the production of FUP or terpestacin by taxonomically diverse plant pathogenic fungi and the presence of highly related gene clusters are suggestive of such a role. Unfortunately, the sequenced reference strain NRRL 62905 used in this study produces fairly low amounts of FUP in vitro compared to other isolates. Disruptions leading to loss of FUP production in other high-level FUP producing strains and strains isolated from different plant hosts, such as tomato [37] or onions [38], seems warranted for virulence testing on the respective hosts. Likewise, the effect of the loss of function of FUP on the fungus-fungus interaction can now be tested. Up to now, FUP is not commercially available and the studies on the mode of action (particularly in plants) of this emerging mycotoxin are limited. For future projects, genetic engineering of a FUP production strain (e.g., by manipulation of regulatory genes and disruption of genes for abundant pigments interfering with purification) should allow the production of analytical standards as well as sufficient toxin for activity tests on plants and fungi. The newly identified side product (compound 11) that seems to be abundant also in the wild type warrants further studies. The identified cluster genes should be useful for the development of PCR tests allowing the monitoring of FUP producing Fusarium strains in maize and other cereal products.

## 4. Material and methods

### 4.1. Strains

*Fusarium proliferatum* NRRL 62905, kindly provided by Dr. Robert Proctor (USDA Peoria IL), was sequenced with DFG funding [22] by BGI Tech Solutions (Hong Kong) Co., Limited. The genome sequence was annotated and previously displayed in the Pedant database at MIPS (not maintained) and can be accessed in Ensemble Fungi (https://fungi.ensembl.org/Fusarium_proliferatum_gca_900029915/Info/Index, accessed on 19 May 2021).

### 4.2. Preparation of Knock-Out Strains 

For the disruption of candidate FUP genes, all flanking regions were amplified from genomic DNA and cloned (5´ region BcuI/SfiI; 3´ region HindIII/SalI) into the vector pKT245 flanking the resistance cassette. The primers used for the amplification are listed in Table 2. The fragments for the transformation were obtained by digestion of the respective plasmid. The transformations were performed as described [26]. Candidates, which were able to grow on regeneration media supplemented with 30 mg/L G418, were screened by PCR using one primer outside the flanking region used for homologous recombination, one primer located within the resistance cassette and the third one in the gene which should be disrupted. The primers located in the resistance cassette were cbh2-E (5´-GAGCATGAGCCTATGGCGATCAGT-3´) and HSVtk-F (5´-GCCACAGCAGCCACGACA-3´). For screening of the knock-out of *FUP1*, multiplex PCR was performed. In this case, three primers (A, E, C-5´, B, F, D-3´) were added in the PCR mix. The disruption of the other genes was screened separately for the wild-type and the knock-out fragments. Primary transformants were purified by sporulation, second-generation strains derived from single spore cultures were used for further analysis.

### 4.3. Toxin Test and Extraction of Secondary Metabolites

For the toxin production tests, 2 g rice were soaked for one hour with 2 mL water in 50 mL Greiner tubes with a foam plug, followed by autoclaving. The rice media were inoculated with 1 × 10^5^ conidia and the cultures were incubated at 20 °C for two weeks in the dark. Three replicas were prepared for each time point. Prior to extraction, the rice cultures were stored at −20 °C. Per g of rice, 4 mL of the extraction solvent methanol was added, followed by crushing with a spatula and homogenization using an Ultra Turrax® (IKA T 25). The samples were incubated for one hour at 20 °C with shaking at 140 rpm, followed by centrifugation at 4000 rpm, 4 °C for 5 min. One mL of the supernatant was transferred to a 1.5 mL tube, which was centrifuged at 20,238 g for 5 min. The samples were measured by LC-MS/MS as previously described [39].

### 4.4. LC-HRMS/MS-Based Target Screening and Suspect Analysis of Intermediates of Fusaproliferin Biosynthetic Pathway

#### 4.4.1. Chemicals and Standards

Acetonitrile (ACN, LC-MS CHROMASOLV^®^), Methanol (MeOH, LC-MS CHROMASOLV^®^), and formic acid (FA, MS grade, ~98% purity) were purchased from Riedel-de Haën, Honeywell (Seelze, Germany). The ultra-pure water was obtained from an ELGA Purelab system Veolia Water (Ultra AN MK2, Vienna, Austria). A standard solution mixture of FUP and terpestacin used for the LC-HRMS/MS measurement was prepared in the solvent mixture ACN/MeOH/H_2_O (1/1/2) + 0.1% FA in the concentration of 20 mg/L of each.

#### 4.4.2. Purification Fusaproliferin and Terpestacin

Both compounds were purified from rice cultures (10 g) inoculated with 5 × 10^5^ conidia of a high producing Austrian isolate (*F. proliferatum* strain 2015-21). To avoid intensive pigment formation two-week-old cultures were extracted with 10-fold volumes of ethyl acetate, shaken for 60 min on a rotary shaker and centrifuged. After evaporation of the extraction solvent using a rotary evaporator, the remainder was taken up in methanol, centrifuged again and purified with an 1100 series preparative HPLC system (Agilent, Waldbronn, Germany). A Gemini NX preparative column (Phenomenex, Torrance, CA, USA) of 21 × 150 mm (5 µm particle size) was used for separation. Mobile phases were ultra-pure water (phase A) and methanol (phase B). The column was equilibrated with 50% B, which was also held for 1 min after injection. A steep gradient up to 100% B followed until minute 5, prior to a wash period with 100% B until minute 10 and re-equilibration with 50% B. In total, 13 time-based fractions were collected between 5.0–7.5 min for each run. Each fraction was tested by LC-MS and fractions containing terpestacin and fusaproliferin were pooled. Methanol from the pooled fractions was removed on a rotary evaporator and the aqueous solutions were lyophilized. In total 3.5 mg fusaproliferin and 1.1 mg terpestacin of high purity were obtained and used as analytical standards for consecutive LC-MS measurements. The calibration standard dilution mix containing terpestacin and fusaproliferin were prepared in the following concentrations: (5, 20, 30 and 50) mg/l in a monophasic solvent mixture containing MeOH/H_2_O (1/1) + 0.1% FA.

#### 4.4.3. LC-HRMS/MS Measurement

The samples and the standard mixture were measured via LC-HRMS(/MS) on the orbitrap mass spectrometer (QExactive, Thermo Fisher Scientific, Bremen, Germany) which is coupled to a Vanquish uHPLC system (Thermo Fisher Scientific, Bremen, Germany).

##### Liquid Chromatography

Reversed-phase chromatography was performed with a C18 analytical column (XBridge®, 3.5 µm × 2.1 × 150 mm, Waters, Milford, Torrance, CA, USA) equipped with a C18 pre-column (4 × 3 mm, Phenomenex, Aschaffenburg, Germany). The gradient method was adapted from Neumann, et al. [40] using H_2_O + 0.1% FA as eluent A and MeOH + 0.1% FA as eluent B with a flow rate of 250 µl/min. While the samples were kept at 10 °C in the autosampler, the column was thermostated to 25 °C.

##### Mass Spectrometry

Full MS measurements were performed in polarity switching mode while ddMS2 measurements were acquired in positive ionization mode. The ionization in positive mode was achieved while applying +3.5 kV and −3.5 kV in negative mode on the heated needle (320 °C) in the HESI source. The scan range was set between *m/z* 100–1500 for full MS and *m/z* 100–1000 for the MS1 level of ddMS2. The resolving power of 120,000 (FWHM at 200 *m/z*) was used for both measurement types (full MS and MS1 of ddMS2). In the ddMS2, a stepped collision energy was applied (25, 35 and 45 eV) for fragmentation and the MS2 were acquired at resolving power of 45,000 FWHM at 200 *m*/*z*. The inclusion list used for the fragmentation contained theoretically calculated *m*/*z* of varying adduct ions of compounds from the terpestacin biosynthetic pathway proposed in Narita et al. [27] as well as of those that are assigned as putatively interesting compounds. The list of target and suspect compounds is given in Table 4.

#### 4.4.4. Feature-Based Molecular Networking

Raw files were converted to .mzML using msConvert (version information in Table 5) [41]. The .mzML files were then imported to XCMS [42] and processed according to step 3 of the GNPS “FBMN with XCMS” documentation (https://ccms-ucsd.github.io/GNPSDocumentation/featurebasedmolecularnetworking-with-xcms3/, accessed on 21 September 2020). CentWaveParam peakwidth was set to 2–50 s, and the signal-to-noise threshold was set at 10. PeakGroupsParam minimum fraction was changed to 0.85. PeakDensityParam minimum fraction was set to 0.4 and the bandwidth was set to 20. All other parameters were left at default. CAMERA annotation (substep 6) was skipped, and export option A was used.

Files were transferred to GNPS via FTP. Feature-based molecular networking [29,43] was performed. The precursor mass tolerance was set to 0.2, and the fragment ion mass tolerance was 0.5. Under advanced network options, the minimum cosine score to be considered a pair was changed to 0.5 and minimum matched fragment ions was changed to 5. The maximum shift between precursors was lowered to 93, the largest theoretical difference between the *m*/*z* values of the compounds of interest. The network top k was increased to 20 in order to see as many connections as possible. No library search was performed, and all other parameters were left as default.

## Figures and Tables

**Table 1:** Structure of the FUP-cluster in *F. proliferatum* strains NRRL 62905 and ET1.

Gene Designation	Annotation	NRRL 62905 Gene Protein	ET1 Gene Protein	Protein Identity
	Prolyl-tRNA synthetase	FPRN_05483 (CVK87167) *	FPRO_05644 CZR39164	Diff. N-terminus 217/365 (59.5%)
*FpFUP2*	Cytochrome P-450 monooxygenase (CYP65A1)	FPRN_05484 CVK87169	FPRO_05645 CZR39163	506/509 (99.4%)
*FpFUP1*	Geranylgeranyl diphosphate synthase, TPS	FPRN_05485 CVK87172	FPRO_05646 CZR39162	744/753 (98.8%)
*FpFUP4*	FAD linked oxidase, N-terminal	FPRN_05486 CVK87175	FPRO_05647 CZR39161	506/519 (97.5%)
*FpFUP5*	Acyltransferase-related	FPRN_05487 CVK87178	FPRO_05648 CZR39160	375/390 (96.2%)
*FpFUP3*	Cytochrome P450	FPRN_05488 CVK87181	FPRO_05649 CZR39159	521/529 (98.5%)
(*FpFUP6*)	Transcription factor domain, fungi	FPRN_05489 CVK87185	FPRO_05650 CZR39158	694/700 (99.1%)
(*FpFUP7*)	Zn(II)2Cys6 transcription factor	FPRN_05490 CVK87187	FPRO_05651 CZR39157	235/245 (95.9%)
	Lactate/Malate dehydrogenase	FPRN_05491 CVK87189	PRO_05652 CZR39156	318/325 (97.8%)

* Problematic gene model.

**Table 2:** Primers used for amplification of flanking regions of FUP genes.

Fragment	Primer (5´–3´)	Length (bp)
FUP1-5´	TAATGGCCGCATAGGCCGCGTCAACAGCTGCTCCC	620
	AAACTAGTCGGTTAGTCTTTGCGAGCTCTCC	
FUP1-3´	AATGTCGACGCCTCATTTACACGGACTACAAGGTC	593
	ACAAGCTTGAAATGTGCAGCATGGCGGTG	
FUP2-5´	TAATGGCCGCATAGGCCTTGCATTATCCTGTTACCTTACTC	640
	AAACTAGTCGGTTAGTCTTTGCGAGCTCTCC	
FUP2-3´	AATGTCGACGCCTCATTTACACGGACTACAAGGTC	576
	ACAAGCTTGAAATGTGCAGCATGGCGGTG	
FUP3-5´	TAATGGCCGCATAGGCCGCAGGTTTAGCTCTGCCTGTAGG	732
	AAACTAGTCAAGTCGTCTTGAGATGATGACAAGCTC	
FUP3-3´	AATGTCGACGCTCTTTGAGACGACCGCTG	599
	ACAAGCTTCGAGGCAGAAGAGGGAGTAACC	
FUP4-5´	TAATGGCCGCATAGGCCGTAAACGCCACCATGCTCAGC	650
	AAACTAGTGCGAAACCAAGGATTCTGAATGCCG	
FUP4-3´	AATGTCGACGTTCACGACAGCCACCTTCAGG	671
	ACAAGCTTGACCGCATTCATAATTGGGCAA	
FUP5-5´	TAATGGCCGCATAGGCCGCCGTTGAATTACCCAGATCCCA	609
	AAACTAGTGTTGAGAGTACAGTACAGGTGCAATG	
FUP5-3´	AATGTCGACCCTGAGCAGATTCTGGAACCGC	539
	ACAAGCTTCCTCATATCCACAACAATACACTAGATTGCC	

**Table 3:** Primers used to obtain disruption specific fragments (5’–3’).

Region	Outside (5’–3’)	Gene specific (5’–3’)
FUP1-5´	GATGGGGCGTCAGTTCTTGAGATCG	CCGTCTCTTGAGCTTCCTCTGCC
FUP1-3´	CGCCCTATCAAGCCAGAGTGCC	GAGCACAGCTACGGAGACACCAG
FUP4-5´	GCATAACGACCTCTCTATAGG	GGAGCAAGGAGGACACTGG
FUP4-3´	CGCACCTGGACTTGTGACC	GTCACGTCACTATCGGACCTTG
FUP5-5´	CGTCGGCAACTGACCCTAACC	CAAGACCAGAATCGGAGTAGTGAGC
FUP5-3´	CAAGATCTAATGGTGATCTTC	GTCCACCCAGTAAACGGACCTTG
FUP3-5´	GCGTCTGACATGCCGAGAGATG	CCATCGAACGGAAATGGTATTGGCC
FUP3-3´	CGTTCTGGACTTTGCTGGGTAC	GGGTTTACGGGACGGAGGAAG

**Table 4:** List of suspected and identified compounds. Chromatograms are shown in Figure 2. Ion species presented in bold were used for the semi-quantitative analysis (Figure 3). Ion types in blue were used for the molecular networking analysis (Figure 4).

Nr.	Molecular Formula	Monoisotopic Mass	Retention Time	Adduct Type	*m*/*z*	Compound Annotated/Identified
1	C_25_H_42_O	358.3236	34.28	[M−H_2_O+H]^+^	341.3203	Preterpestacin I
				**[M+H]^+^**	359.3308	
				[M+Na]^+^	381.3128	
				[M+NH_4_]^+^	376.3574	
2	C_25_H_42_O_2_	374.3185	32.44	[M−H_2_O+H]^+^	357.3152	Preterpestacin II a
				[M+H]^+^	375.3258	
				**[M+Na]^+^**	397.3077	
				[M+NH_4_]^+^	392.3523	
				[M+HCOO]^−^	419.3167	
				[M+Cl]^−^	409.2868	
3	C_25_H_40_O_2_	372.3028	33.05	[M−H_2_O+H]^+^	355.2995	Preterpestacin II c
				[M+H]^+^	373.3101	
				[M+Na]^+^	395.2921	
				**[M+NH_4_]^+^**	390.3367	
4	C_25_H_40_O_3_	388.2977	32.15	[M−H_2_O+H]^+^	371.2945	Preterpestacin II b
				[M+Na]^+^	411.2870	
				**[M−H]** ^−^	387.2905	
				[M+HCOO]^−^	433.2959	
				[M+Cl]^−^	423.2660	
5	C_25_H_42_O_4_	406.3083	27.02	[M−H_2_O+H]^+^	389.3050	Preterpestacin III
				[M+Na]^+^	429.2975	
				**[M+HCOO]^−^**	451.3065	
6	C_25_H_40_O_4_	404.2927	26.4	[M−H_2_O+H]^+^	387.2894	Two tautomers keto or enol
				[M+Na]^+^	427.2819	
				**[M−H]^−^**	403.2854	
				[M+HCOO]^−^	449.2909	
7	C_25_H_38_O_4_	402.2770	27.73	[M−H_2_O+H]^+^	385.2737	Terpestacin keto from
				[M+Na]^+^	425.2662	
				**[M−H]^-^**	401.2697	
				[M+HCOO]^−^	447.2752	
				[M+Cl]^−^	437.2453	
8	C_25_H_38_O_4_	402.2770	26.6	[M−H_2_O+H]^+^	385.2737	**Terpestacin** **enol form** **TPC**
				**[M+H]^+^**	403.2843	
				[M+Na]^+^	425.2662	
				[M−H]^−^	401.2697	
				[M+HCOO]^-^	447.2752	
				[M+Cl]^−^	437.2453	
9	C_25_H_36_O_4_	400.2614	26.87	[M−H_2_O+H]^+^	383.2581	Terpestacin aldehyde
				**[M+H]^+^**	401.2686	
				[M−H]^−^	399.2541	
10	C_27_H_40_O_5_	444.2876	27.57	[M−H_2_O+H]^+^	427.2843	**Fusaproliferin** **FUP**
				**[M+H]^+^**	445.2949	
				[M+Na]^+^	467.2768	
				[M+NH_4_]^+^	462.3214	
				[M−H]^−^	443.2803	
11	C_25_H_40_O	356.3079	34.2	[M−H_2_O+H]^+^	339.3046	Oxo-preterpestacin I
				**[M+H]^+^**	357.3152	
				[M+Na]^+^	379.2971	

**Table 5:** Version numbers used for individual packages.

Package	Version
msConvert [41]	ProteoWizard 3.0.20140 (aaf841559)
R [44]	3.6.3
XCMS [42]	3.8.0
pander	0.6.3
GNPS [29,43]	Workflow version release_25

## Data Availability

Not applicable.

## Supplementary Materials

The following are available online at https://www.mdpi.com/article/10.3390/toxins13070468/s1: Figure S1: Supplementary Overview of the fragmentation spectra used for the molecular networking in Figure 4, Figure S2: Supplementary Overview of the fragmentation spectra used for the molecular networking in Figure 4, Figure S3: Supplementary Overview of the fragmentation spectra used for the molecular networking in Figure 4, Figure S4: *m/z* of intense peaks commonly found in the MS2 of all target and suspect compounds.
