# Supplementary material for: Identification and Functional Characterization of the Gene Cluster Responsible for Fusaproliferin Biosynthesis in Fusarium proliferatum
Source: Toxins (Basel). 2021 Jul 6;13(7):468. doi: 10.3390/toxins13070468 (PMC8310001; doi:10.3390/toxins13070468)
Supplement: Supplementary file 1 [file toxins-13-00468-s001.zip › toxins-1196347-supplementary update.pdf]

Supplementary Materials: Identification and functional characterization of the gene cluster responsible for fusaproliferin biosynthesis in *Fusarium proliferatum*

Asja Ćeranić, Thomas Svoboda, Franz Berthiller, Michael Sulyok, Jonathan Samson, Ulrich Güldener, Rainer Schuhmacher and Gerhard Adam

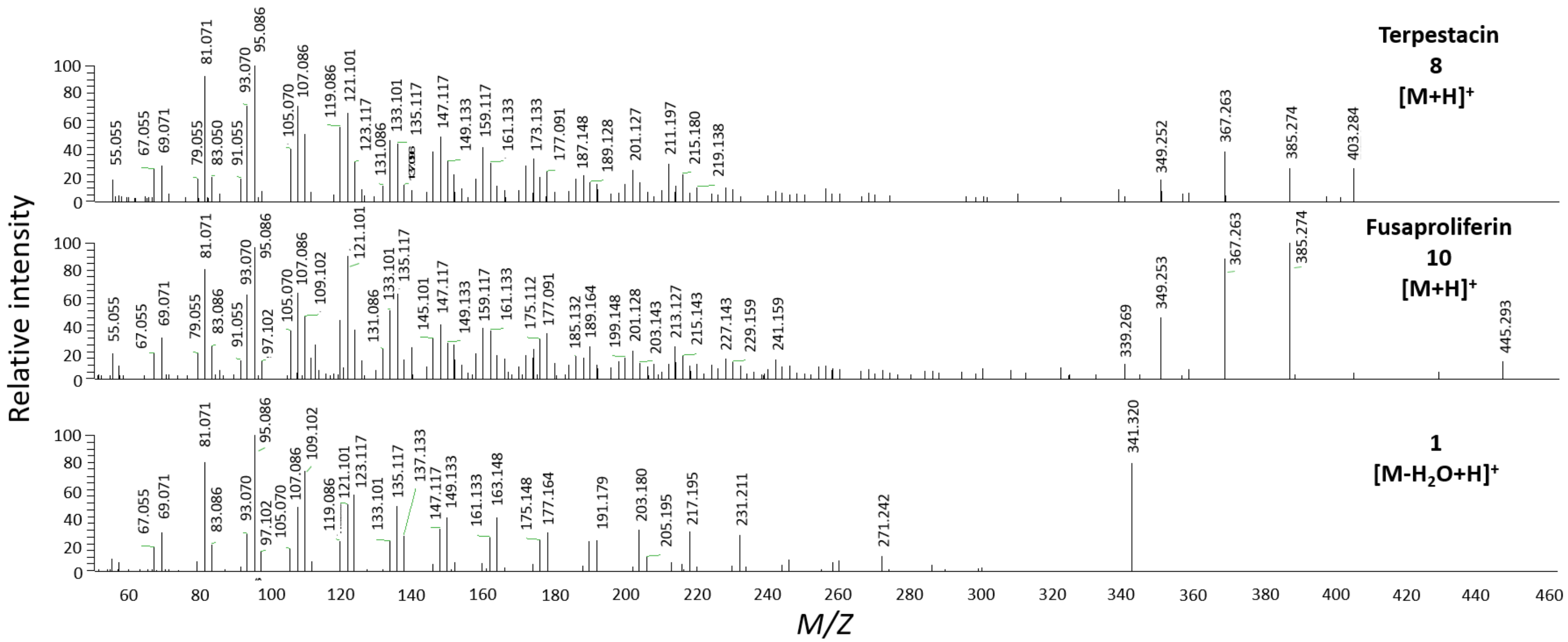

Figure S1. Supplementary Overview of the fragmentation spectra used for the molecular networking in **Figure 4.**

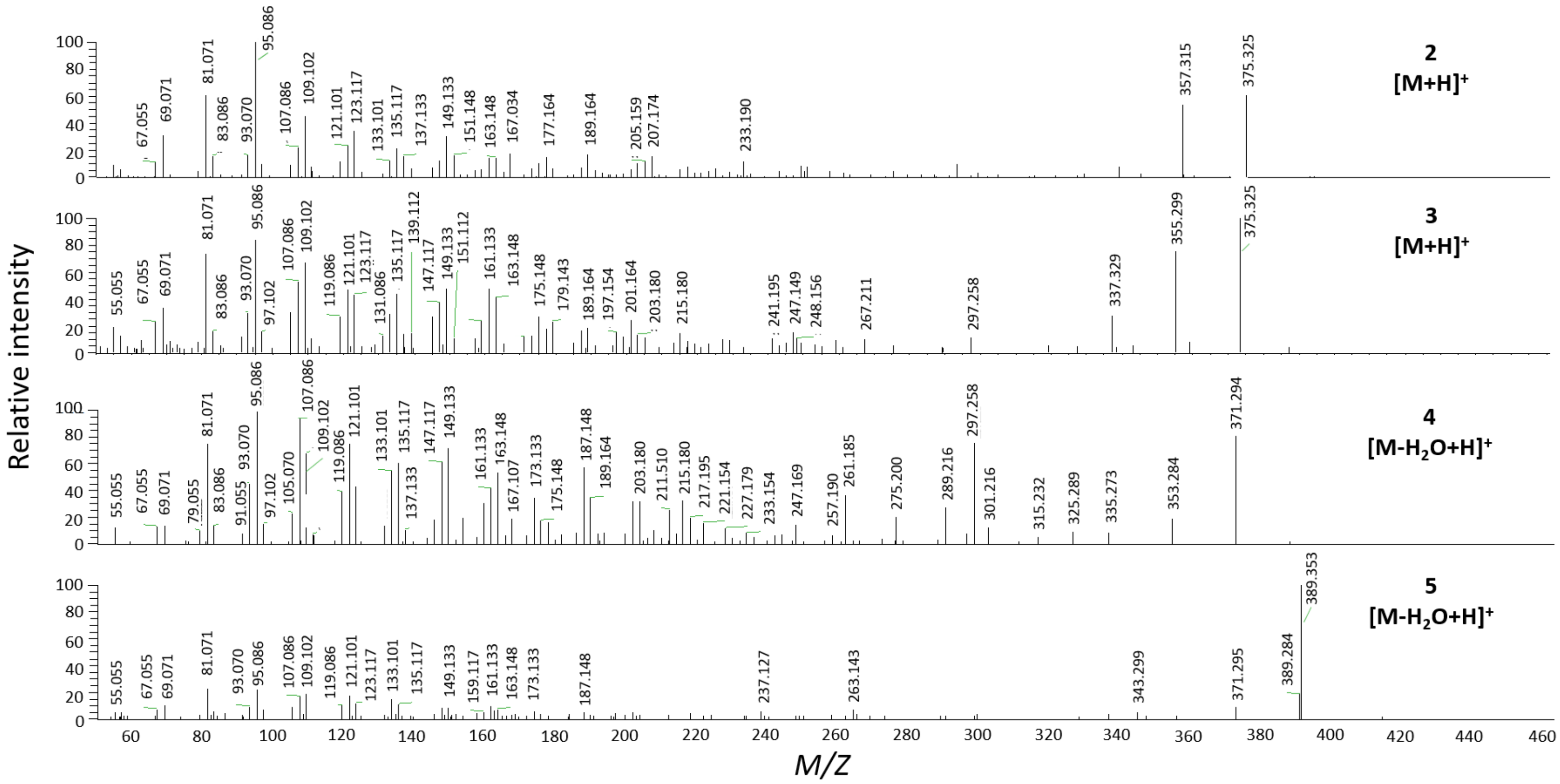

Figure S2. Supplementary Overview of the fragmentation spectra used for the molecular networking in **Figure 4.**

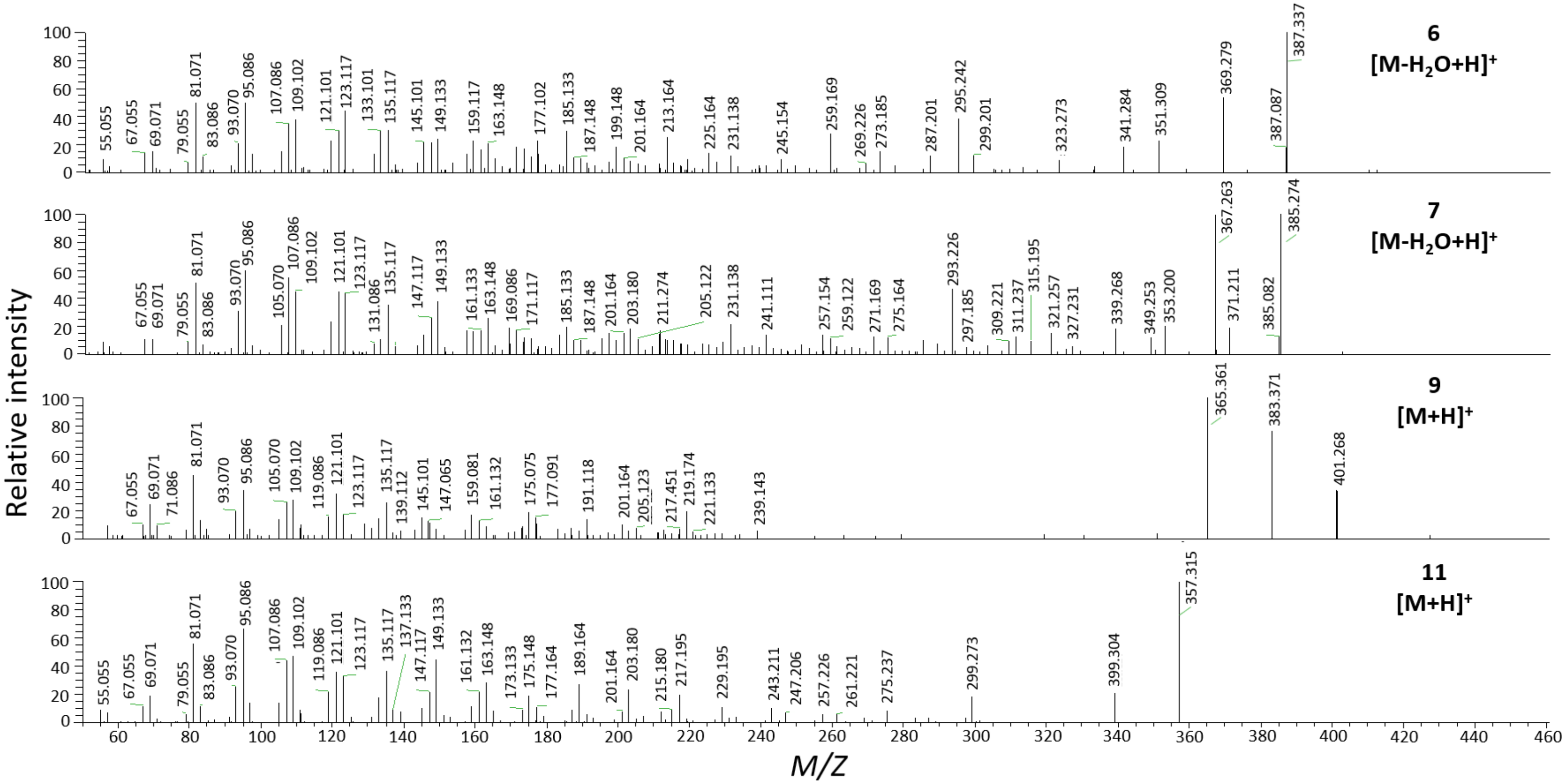

Figure S1. Supplementary Overview of the fragmentation spectra used for the molecular networking in **Figure 4.**

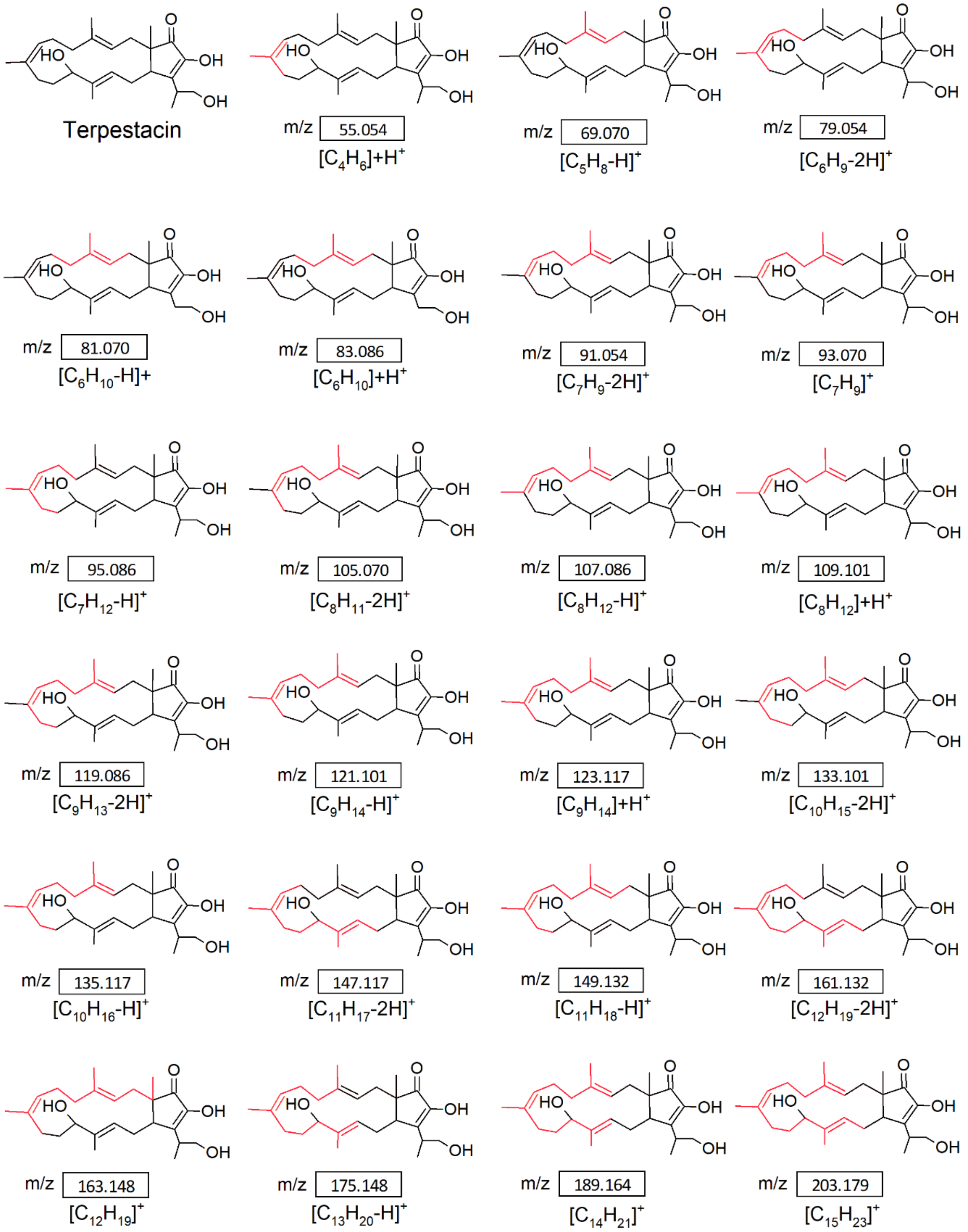

Figure S4. m/z of intense peaks commonly found in the MS2 of all target and suspect compounds. On the example of terpestacin (TPC), the fragment formula for the respective m/z was generated via MetFrag and those structure parts which match the fragment formula are represented in red.
